# Midterm survival of imaging-assisted robotic lung segmentectomy for non-small-cell lung cancer

**DOI:** 10.1093/icvts/ivab287

**Published:** 2021-10-23

**Authors:** Zied Chaari, François Montagne, Matthieu Sarsam, Benjamin Bottet, Philippe Rinieri, Andre Gillibert, Jean Marc Baste

**Affiliations:** 1 University of Sfax—Department of Thoracic and Cardiovascular Surgery, Habib Bourguiba University Hospital, Sfax, Tunisia; 2 Department of General and Thoracic Surgery, Rouen University Hospital, Normandy, France; 3 Department of Epidemiology and Public Health, Rouen University Hospital, Normandy, France; 4 INSERM U1096, Rouen University Hospital, Normandy, France

**Keywords:** Robotic surgery, Segmentectomy, Non-small-cell lung cancer, Midterm survival

## Abstract

**OBJECTIVES:**

Our goal was to report our midterm results using imaging-assisted modalities with robotic segmentectomies for non-small-cell lung cancer (NSCLC).

**METHODS:**

This was a retrospective study of all robotic segmentectomies, with confirmed NSCLC, performed at our general and thoracic surgery unit in the Rouen University Hospital (France), from January 2012 through December 2019. Benign and metastatic lesions were excluded. Data were extracted from the EPITHOR French nationwide database.

**RESULTS:**

A total of 121 robotic segmentectomies were performed for 118 patients with a median age of 65 (interquartile range: 60, 69) years. The majority had clinical stage T1aN0M0 (71.9%) or T1bN0M0 (13.2%). The mean (standard deviation) number of resected segments was 1.93 (1.09) with 80.2% imaging-assisted segmentectomies. Oriented (according to tumour location) or systematic lymphadenectomy or sampling was performed for 72.7%, 23.1% and 4.1% of patients. The postoperative course was uneventful for 94 patients (77.7%), whereas 34 complications occurred for 27 patients (22.3%), including 2 patients (1.7%) with Clavien-Dindo ≥III complications. The mean thoracic drainage duration was 4.12 days, and the median hospital stay was 4 days (interquartile range: 3, 5) after the operation. The 2-year survival rate was 93.9% (95% confidence interval: 86.4–97.8%). Excluding stage IV (*n* = 3) and stage 0 tumours (*n* = 6), the 2-year survival rate was 95.7% (95% confidence interval: 88.4–98.8%) compared to an expected survival rate of 94.0% according to stage-specific survival rates found in a large external reference cohort.

**CONCLUSIONS:**

Imaging-guided robotic-assisted thoracic surgery segmentectomy seems to be useful and oncological with good midterm results, especially for patients with early-stage NSCLC.

**Figure:**
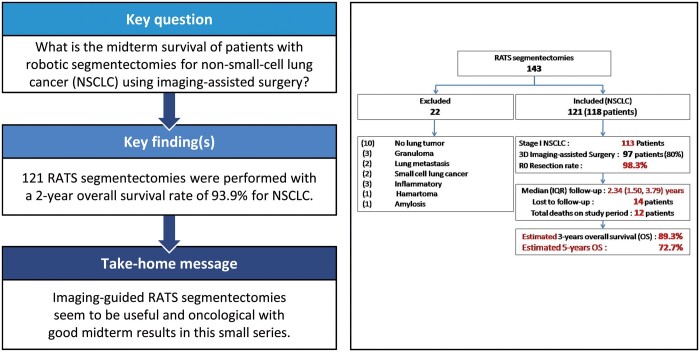

## INTRODUCTION

Anatomical resection is the standard treatment for early-stage non-small-cell lung cancer (NSCLC). Lobectomy with complete lymphadenectomy remains the gold standard for treatment of early-stage NSCLC. Since the first randomized trials in 1995 [1, 2] comparing anatomical lobectomy versus sublobar resection for stage I, lung segmentectomies have been proposed for elderly patients with impaired lung function or for those who cannot support lobectomy, with good short- and long-term results.

In 2002, Melfi *et al.* [3] reported the first robotic-assisted thoracic surgery (RATS) with 5 lobectomies. Since then, several series have been published about first experiences with short-term outcomes for RATS segmentectomies [4–7], but few authors have reported long-term outcomes.

The goal of our study was to report our short-term and midterm results for NSCLC in patients operated on by RATS segmentectomies, including mostly interventions with 3-dimensional imaging reconstructions.

## MATERIALS AND METHODS

### Study design

This study was a prospective cohort study of patients operated on in the General and Thoracic Surgery Unit at Rouen University Hospital (France). All patient data were recorded in the nationwide EPITHOR registry since the introduction in 2012 of the daVinci Si robotic platform (Intuitive Surgical, Sunnyvale, CA, USA).

### Inclusion and exclusion criteria

We included all patients who underwent RATS segmentectomies for confirmed NSCLC from January 2012 to December 2019. We excluded patients who underwent RATS for lung metastasis, benign lesions and resection other than segmentectomy (wedge or other resections).

### Preoperative management

Pathological diagnosis was carried out either preoperatively [bronchoscopy or computed tomography (CT)-guided biopsy] or postoperatively. Segmentectomy was planned for patients with limited lung function (forced-expiratory volume in 1 s <60%), slowly evolving lesions or ground-glass lesions <2 cm.

Preoperative assessment included a recent CT scan (dating <5 weeks), a positron emission tomography scan, brain imaging (scan or magnetic resonance imaging) and pulmonary function tests.

**Figure 1:**
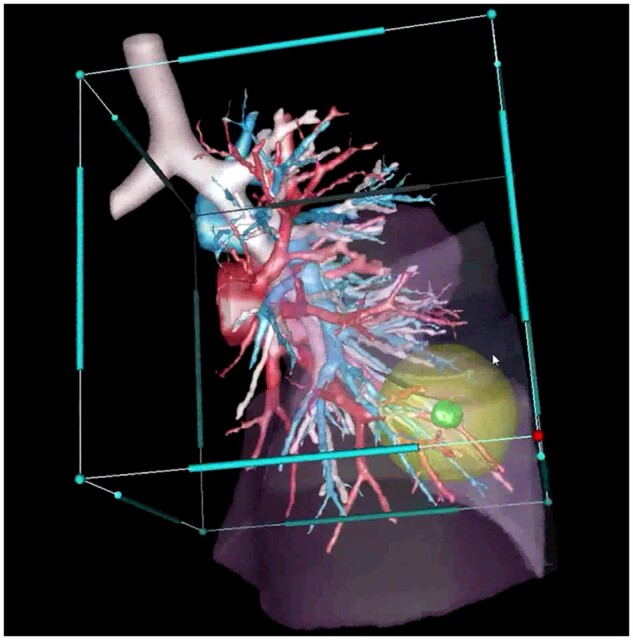
Multimodal reconstruction imaging system showing tumour with 2-cm free margins (left S9S10 segmentectomy).

Since 2015, all preoperative planning of RATS segmentectomies was based on a multimodal reconstruction imaging system (Visible Patient, Strasbourg, France), and viewed with Anywhere-Imaging Software (Therapixel, Valbonne, France). Three-dimensional (3D) models enable visualization of bronchi and of the divisions of pulmonary vessels and anticipation of oncological margins (Fig. 1).

### Surgical technique

We performed all segmentectomies with lymphadenectomies according to the international guidelines for operable NSCLC [8–10].

As described previously [11], we started our robotic programme in 2012 and switched to the daVinci X platform in July 2018. We started to perform a 3-arm technique (12 mm for a camera, 8.5 mm for robotic endowrist instruments and an assistant trocar in the 9th or 10th intercostal space for suction and stapling) [12]. All robotic procedures (lung resection and mediastinal surgery) were performed with CO_2_ insufflation (5–8 mmHg, depending on the patient’s tolerance) in order to enhance the working space in the pleural cavity.

We then moved to a 4-arm technique [13] using robotic staplers. Before stapling, we ventilate the lung after clamping the target segment’s bronchus. The inter segmental plane was demarcated using electrocoagulation before stapling. The new X platform allowed us to use invisible near-infra-red fluorescence imaging with indocyanine green (ICG) to detect and localize pulmonary tumours and inter-segmental planes [14].

If the lesion was close to the inter-segmental plane, a double endobronchial dye marking was done by an interventional pulmonologist before the operation. The blue and green mixture around the lesion provided visual representation of surgical margins at all times.

**Figure 2:**
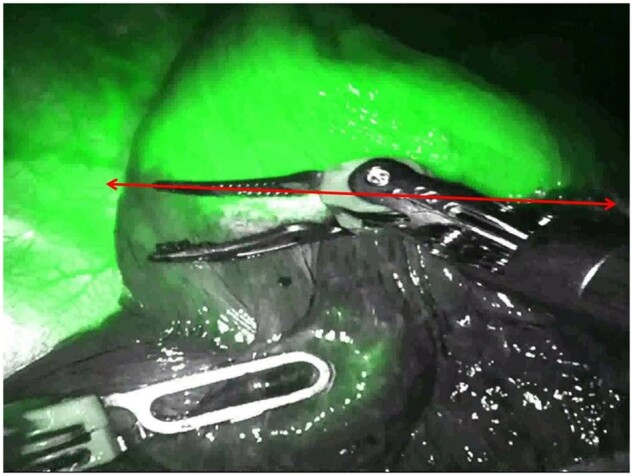
Inter segmental plane identification (red line) after administration of indocyanine green.

During the operation, we embedded the SPY imaging system (Stryker, Kalamazoo, MI, USA) to localize the lung tumour. The inter-segmental plane was localized by ICG injection (20 ml/25 mg) (Fig. 2) before stapling [15, 16]. If an air leak was detected, a surgical sealant was applied on the staple lines [17].

### Perioperative management

All patients were evaluated by anaesthesiologists. Basic blood tests, electrocardiograms, chest imagery and pulmonary function tests were performed according to their comorbidities.

After the operation, pain management was achieved through loco-regional nerve block (serratus plane, intercostal or paravertebral), and the Enhanced Recovery after Surgery protocol was applied to all patients (early ambulation, introduction of food, switching from intravenous to oral drugs and keeping morphine painkillers to a minimum).

### Data collection and monitoring

All patient data were recorded in the French Nationwide Database (EPITHOR)and in local medical records, from 2012 and ongoing. Operative data included the side, surgery duration, number of trocars, perioperative bleeding, number and location of resected segments and reasons for conversions (if any). Postoperative data included histopathological diagnosis, tumour staging, chest drainage and hospital duration, complications and deaths occurring within 90 days postoperatively. EPITHOR was monitored by our research technician for exhaustivity and data quality.

### Follow-up

Follow-up was performed by physicians and thoracic surgeons according to our usual clinical practice: clinical examination every 3 months for the first 2 years, with a CT scan if needed, then every 6 months for up to 5 years. After 5 years, either annual surveillance or cessation of follow-up was adopted. Follow-up data were obtained via medical records, letters or phone calls to the patients’ practitioners or to the patients themselves if needed. In addition, vital status was ascertained by accessing the national electronic nominative registry of deaths. The latter does not contain data about cause of death and live subjects, making it impossible to differentiate between living or dead subjects who have a typo in the first or last name. Follow-up ended 1 April 2020. Patients lost to follow-up were defined as subjects without news for at least a quarter of the follow-up time and for at least 4 months.

### Outcomes

Outcome variables included overall survival (OS), disease-free survival, 90-day mortality and postoperative complications.

### Statistical analyses

Continuous variables were described by their medians and quartiles. Categorical variables were described by their frequency and percentages. Survival rates and curves were computed using the Kaplan–Meier method, and their confidence intervals (CIs) were computed by the beta product confidence procedure, which is a generalization of the Clopper–Pearson CI for censored data (valid even when there are zero events). On large samples, it is equivalent to Greenwood’s method, which is used together with the Kaplan–Meier estimator in most publications. Death rates were computed as 1 minus the survival rates.

The 2-year survival rate was compared to that of a large external cohort (*n* = 3950 patients) that was used to compare AJCCv8 to AJCCv7 [18]. We performed graphical extraction of the 2-year death rates by AJCCv7 stage from Yun *et al.*’s figures [18]. Then, we computed the expected 2-year survival rate in our cohort, excluding stages 0 and IV because survival rates were not described in that article. This theoretical survival rate was informally compared to the survival rate CI in our cohort.

Medians and quartiles of the clinical follow-up were estimated by the Brookmeyer-Crowley method. Similarly, the inverse Kaplan–Meier estimator was used to estimate censorship rates at 6 months and at 1, 3, and 5 years.

Missing clinical TNM stages (*n* = 15) were simply imputed by pathological TNM stages, and, conversely, missing pathological TNM stages (*n* = 5) were simply imputed by clinical TNM stages.

Although multiple-intervention patient outcomes were not independent, this correlation was neglected.

Survival analyses were performed with R statistical software (The R Foundation for Statistical Computing, Vienna, Austria).

## RESULTS

During the study period, 989 surgical resections for NSCLC were performed, including 91 with open surgery. A minimally invasive approach was used to perform 25 pneumonectomies, 23 bilobectomies, 675 lobectomies and 175 segmentectomies including 121 RATS segmentectomies, performed for 118 patients by 4 different surgeons. The RATS proportion in the minimally invasive segmentectomies [video-assisted thoracoscopic surgery (VATS) and RATS] increased from 7/20 (35.0%) during 2012–2014, to 40/46 (87.0%) during 2015–2016 and 74/102 (72.5%) from 2017 (*P* = 0.051 for trend). One surgeon performed 98 (81.0%) interventions, the second performed 16 (13.2%), the third 6 (5.0%) and the fourth 1 (0.8%). Patient characteristics are listed in Table 1.

**Table 1:** Patient characteristics

Variable	Frequency (%) or median (IQR)
Total	*N* = 121
Age in years	66 (60, 70)
Male sex	70 (57.9)
ECOG performance status	
0	89 (73.6)
1	30 (24.8)
2	2 (1.7)
Former or current smoker	89 (73.6)
% of theoretical forced expiratory volume in 1 s	92 (76, 100), *n* = 110^a^
% of theoretical forced expiratory volume in 1 s < 60%	10 (9.1), *n* = 110^a^
Carbon monoxide diffusing capacity	66 (60, 80), *n* = 43^a^
Neoadjuvant therapy	5 (4.1)
Comorbidities	
History of other cancer	53 (43.8)
Other pulmonary cancer	16 (13.2)
Hypertension	29 (24.0)
Chronic pulmonary disease (asthma, COPD, chronic bronchitis)	27 (22.3)
Severe obesity	5 (4.1)
Diabetes	7 (5.8)
Dyslipidaemia	7 (5.8)
Congestive heart failure	3 (2.5)
Robotic arms, *n*	
3	86 (71.1)
4	35 (28.9)
Conversions	
Haemorrhage	1 (0.8)
Invasive tumour	1 (0.8)
Pleural symphysis	2 (1.7)
Segmentectomy location	
Right S1	7 (5.8)
Right S2	19 (15.7)
Right S3	3 (2.5)
Right S1–S2	2 (1.7)
Right S2–S6	1 (0.8)
Right S6	9 (7.4)
Right S7–S10	7 (5.8)
Left S2	4 (3.3)
Left S3	3 (2.5)
Left S1–S2	10 (8.3)
Left S2–S3	1 (0.8)
Left S1–S3	25 (20.7)
Left S4–S5	7 (5.8)
Left S6	15 (12.4)
Left S7	1 (0.8)
Left S7–S10	7 (5.8)
Final pathological diagnosis	
Adenocarcinoma	103 (85.1)
Squamous cell carcinoma	10 (8.3)
Carcinoïd tumour	6 (5)
Large-cell carcinoma	2 (1.7)
cTNM tumour staging	
T1aN0M0	87 (71.9)
T1bN0M0	16 (13.2)
T1cN0M0	2 (1.7)
T2aN0M0	8 (6.6)
T3N0M0	2 (1.7)
T1aN2M0	2 (1.7)
T4N0M0	1 (0.8)
T4N0M1a	1 (0.8)
T4N2M1b	1 (0.8)
T1bN0M1b	1 (0.8)
Follow-up in days (censored on death)	2.34 (1.50, 3.79)

COPD: chronic obstructive pulmonary disease; ECOG: Eastern Cooperative Oncology Group; IQR: interquartile range; TNM: tumour-node-metastasis.

a Non-missing data (of 121 patients).

No 3D reconstruction was carried out for 24 segmentectomies between 2012 and 2015. This technique was used as part of a research project in 2015. Since then, it has been performed systematically, given the constant discovery of vascular and bronchi variations; overall, 97/121 (80.2%) interventions were performed with 3D reconstruction.

High-stage preoperative clinical stages were explained, respectively, by suspected N2 involvement (*n* = 2), a nodule in the contralateral lung (*n* = 1) and brain metastases (*n* = 2) (Table 2). The mean (standard deviation) number of resected segments was 1.93 (1.09) with a median at 1 [interquartile range (IQR): 1, 3]. The median duration of the operations was 100 min (IQR: 80,130) whereas the mean (standard deviation) operative time was 114 (40) min. Most durations were an exact multiple of 30 min (57.9%) or 10 min (98.3%), suggesting rounding in records.

**Table 2:** Tumour-node-metastasis stage before and after surgery

	cTNM stage	pTNM stage
Stage in situ, *n* (%)	0 (0.0)	6 (5.0)
Stage IA, *n* (%)	105 (86.8)	83 (68.6)
Stage IB, *n* (%)	8 (6.6)	15 (12.4)
Stage IIA, *n* (%)	0 (0.0)	6 (5.0)
Stage IIB, *n* (%)	2 (1.7)	6 (5.0)
Stage IIIA, *n* (%)	3 (2.5)	2 (1.7)
Stage IIIB, *n* (%)	0 (0.0)	0 (0.0)
Stage IV, *n* (%)	3 (2.5)	3 (2.5)
Total	121	121

TNM: tumour-node-metastasis.

Oriented (according to tumour location) or systematic lymphadenectomy or sampling was performed for 88 (72.7%), 28 (23.1%) and 5 (4.1%) patients respectively. A nodal upstaging from N0 to N1 or N2 was noted in 7 patients (5.8%), whereas tumour upstaging was noted in 42 patients (34.7%), 20 of whom had an impact on the global staging (e.g. stage IB–IIA). All lymph node positivity at station 12, 13 or 14 was reported on the final pathological examination. First drainage lymph node frozen section analysis was not done systematically. Two (1.7%) resections were microscopically incomplete. Conversion was necessary for 4 patients (3.3%) because of pleural symphysis (2 cases), haemorrhage (1 case) and a failed tumourectomy (1 case).

The postoperative course was uneventful for 94 (77.7%) patients. We reported 34 complications in 27 patients (22.3%) according to the Clavien–Dindo classification, including 9 grade I, 23 grade II, and 2 grade III (Table 3). In the latter group, 2 patients (1.7%) had 2 or more associated complications. Mean drainage duration was 4.12 days (range: 2–6 days), and the median hospital stay was 4 days (IQR: 3–5). One (0.8%) patient died within 90 days of a probable brain herniation due to a primitive neurological cancer.

**Table 3:** Postoperative complications (Clavien–Dindo classification)

Complication	Grade	*n* (%)
Prolonged air leak	I	7 (5.8)
Bandage allergy	I	1 (0.8)
Atelectasis	I	1 (0.8)
Infection	II	7 (5.8)
Fever	II	2 (1.7)
Urological complication	II	6 (5)
Respiratory failure	II	1 (0.8)
Atelectasis	II	2 (1.7)
Other grade II complications^a^	II	4 (3.3)
Cardiac rhythm disorder	II	1 (0.8)
Haemorrhage	IIIA	1 (0.8)
Haemorrhage	IIIB	1 (0.8)

a Epilepsy, behaviour disorder, renal complication, subcutaneous emphysema.

The median follow-up was estimated at 2.34 (95% CI: 2.02–2.99) years with the first quartile at 1.38 (95% CI: 1.08–1.89) years and a third quartile at 3.82 (95% CI: 3.44–4.18) years. Fourteen patients (11.6%) were lost to follow-up without recent medical information, whereas 12 (9.9%) died and 95 (78.5%) were administratively censored. Of the 12 deaths, 9 occurred during the first 3 years and 11, during the first 5 years of follow-up. Death was secondary to a relapse of the NSCLC in 7 of 11 (64%) patients who died within 5 years following surgery, another cancer (pulmonary or neurological) in 2 (18%) patients, a pulmonary embolism without relapse in 1 (9%) patient and a possible relapse (without histological confirmation) in 1 (9%) patient.

**Figure 3:**
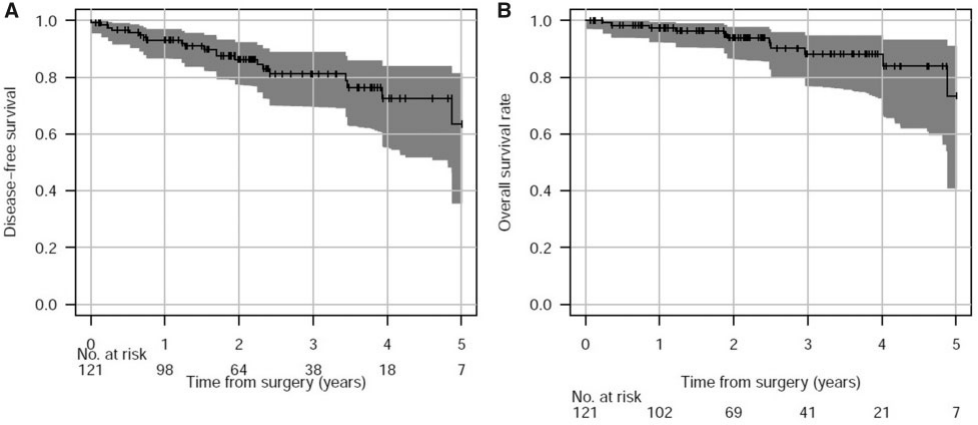
Disease-free survival and overall survival rate curves from the operation for up to 5 years, with 95% confidence bands (grey zone).

The proportion of patients censored at 6 months and at 1, 3 and 5 years was estimated at 5.0%, 13.4%, 61.5% and 92.1%, respectively. The survival curve, including all stages, is shown in Fig. 3. The 2-, 3- and 4-year survival rates were estimated at93.9% (86.4–97.8%), 88.1% (76.8–94.7%) and 88.1% (72.6–94.7%) in the whole cohort (*n* = 121), respectively. When restricted to the 112 patients with stages I to III (excluding stages 0 and IV), the 2-, 3- and 4-year survival rates were estimated at 95.7% (88.4–98.8%), 89.3% (77.1–95.9%) and 89.3% (72.8–95.9%).Taking in account the case-mix of our cohort and stage-specific survival rates at 3 years according to Yun *et al.* [18], the expected 2-year survival rate in patients with stages I to III was 94.0%, inside the CI of the observed survival rate (Table 4).

**Table 4:** Expected survival rated according to stage

pTNM stage	Number of interventions	2-Year death rate, *n* (%, 95% confidence interval)	Expected survival rate (%)
Stage 0	6	1 (20, 0.5–71.6%)	
Stage I	98	2 (2.6, 0.3–10.1%)	
Stage IA	83	2 (3, 0.3–11.9%)	95.25
Stage IB	15	0 (0, 0–33.6%)	88.25
Stage II	12 (2 IIA + 10 IIB)	2 (20.5, 2.5–60.8%)	79.5 (IIA) 71.5 (IIB)
Stage III	2 (2 IIIA)	0 (0, 0–84.2%)	61
Stage IV	3	1 (33.3, 0.8–98%)	
Unknown stage	0	1 (20, 0.5–71.6%)	

p: pathological; TNM: tumour-node-metastasis.

## DISCUSSION

In this monocentric study, we reported our RATS segmentectomy results with image-assisted surgery for NSCLC and our midterm outcomes. Results of major RATS series reported through December 2019 are detailed and compared in this manuscript.

Our study differs from the previous series in several points:

- Firstly, we included a large number of patients who had segmentectomies for early-stage NSCLC, which is the second largest series after that reported by Geraci *et al.* [19].
- Secondly, we reported results of RATS segmentectomies, not only for stage I NSCLC but also for stages II and above.
- Thirdly, our database is monitored by our technician for exhaustivity and data quality.
- Fourthly, we reported a multimodal technique including imaging-assisted resection using 3D lung reconstruction, lung node tracking using dyes and guided inter-segmental plan demarcation.
- Finally, we reported sublobar anatomical resection results with midterm follow-up.

The first anatomical RATS pulmonary resections were described by Melfi *et al.* in 2002 [3]. Since then, several authors have reported their results of anatomical resections for NSCLC or other pathologies. The operative technique used in our department has evolved from the 3-arm to the 4-arm approach as described by Cerfolio *et al.* [4], and 4 different surgeons performed RATS segmentectomies, which differs from the series reported by Toker *et al.* [7], in which he reported single-surgeon results of 102 pulmonary resections including 46 segments.

Today, lobectomy is the main treatment for early-stage NSCLC, whereas segmentectomy is an acceptable alternative. Winckelmans *et al.* compared segmentectomy to lobectomy for stage I NSCLC in a meta-analysis including 28 studies. They found significantly worse OS [hazard ratio (HR) = 1.31, 1.01–1.69] and cancer-specific survival (HR = 1.56, 1.08–2.258) in segmentectomies for stage IA (T1N0M0) than in lobectomies [20]. However, they did not find a significantly worse prognosis for segmentectomies for stage IA < 2 cm tumours (HR = 1.13, 0.86–1.49; *P* = 0.37) compared to lobectomies. Dai *et al.* compared 11520 lobectomies to 769 segmentectomies for AJCCv7 T1aN0M0 NSCLC; they found significantly worse adjusted OS for tumours <1cm (HR = 1.394; 1.013–1.918; *P* = 0.04) and 1- to 2-cm tumours (HR = 1.223, 1.054–1.421; *P* = 0.008). Results for specific survival were similar to those for OS, but survival was worse for wedge resections of 1- to 2-cm tumours [21].

In our study, the median operative time was 100 min (IQR: 80,130) including docking time, with only 4 conversions (3.3%), and there was no significant difference between simple and combined segmentectomies. Surgery duration decreased significantly with the learning curve of the surgeons [11]. In terms of operative duration time, hospital stay and morbidity rates, operative characteristics are comparable with those of other series of RATS segmentectomies (Table 5). We reported the results of RATS segmentectomies for resectable NSCLC, not only for stage I, like other recent publications [22, 23], but also for advanced stages of NSCLC. We believe that selected and fragile patients can benefit from infra lobar resections, which have the advantages of a better anatomical resection and lymph node dissection using a minimally invasive approach compared to the more risky lobectomy.

**Table 5:** Surgical results of published series of robotic-assisted thoracic surgery segmentectomies

Author	Year	Patients (RATS)	Arms	Conversion, *n* (%)	Surgery (min)	Hospitalization (days)	Complications, *n* (%)
Cerfolio *et al.* [4]	2016	100	4	7 (7)	Median (range)	Median (range)	10 (10)
					88 (46–205)	3 (2–9)	
Nguyen *et al.* [23]	2018	71	4	0%	127 (70–227)	Median (range)	29%
						4 (2–31)	
Xie *et al.* [25]	2019	88	4	NR	Mean (SD)	Mean (SD)	11 (12.6)
					116 (22)	5.0 (1.9)	
Zhou *et al.* [22]	2020	50	3 or 4	NR	Mean (SD)	Median (range)	6 (12)
					90 (58)	4 (2–8)	
Geraci *et al.* [19]	2019	245	4	0.8%	Median (range)	Mean (range)	26.5%
					86: (43–250)	3.1 (1–21)	
Our study	2020	121	3 or 4	3.3%	Median (IQR)	Median (IQR)	22.3%
					100 (80, 30)	4 (3, 5)	

IQR: interquartile range; NR: not reported; RATS: robotic-assisted thoracic surgery; SD: standard deviation.

Using a 4-arm approach seemed to be easier than VATS to perform lung resections. RATS allows a better exposure and mimics an open approach for vessels, bronchi and lymph-node dissections. Moreover, we think that RATS has its place for a better lymph node dissection around small vessels and bronchi [5] and in the case of lung adherence in difficult-to-reach areas.

In a meta-analysis including 7438 patients from 14 studies, Liang *et al.* [24] demonstrated that RATS had a significantly lower 30-day mortality rate compared to thoracoscopy. But there were no significant differences between the 2 techniques in terms of postoperative complications, operative time, hospital stay and number of resected lymphnodes. According to Xie *et al.,* compared to thoracoscopy, RATS allowed us to get a greater number of resected lymphnodes with a mean of 13.24 versus 11.71 lymphnodes (*P* = 0.018) [25]. However, the absolute number of lymphnodes resected is a debated oncological quality criterion.

In our department, since 2015, all infra lobar resections were planned and assisted by 3D imaging [26]. In addition, reconstruction software provides a precise description of each anatomical variation for each patient (bronchi, veins, arteries). Moreover, expected margins could be anticipated to perform a “true” oncological and anatomical R0 segmentectomy.

The use of infra-red light with green ICG [19] orients surgeons towards the inter-segmental plane with excellent precision in order to preserve healthy lung parenchyma. It has been demonstrated that a multimodal system including 3D imaging has good operative anatomical accuracy and provides more comfort, helping surgeons to make decisions when they encounter complex situations [27]. Moreover, this technique helped us to perform a picking lymphadenectomy for indolent lesions (ground-glass nodules, lesions smaller than 2 cm, slowly evolving, with low metabolic activity on a positron emission tomography scan) with good results and few long-term recurrences.

Long-term survival after a RATS segmentectomy has been reported in only a few series with small sample sizes. Casiraghi *et al.* [28] reported a series of 29 RATS segmentectomies for early-stage NSCLC with a 5-year survival rate of 96.2%, but with only 4 patients followed up at 6 years. Nguyen *et al.* [23] reported a series of 71 patients with early-stage NSCLC having robotic segmentectomies with a specific 5-year stage I NSCLC survival rate of 73%.

The 5-year OS rate was compared between 406 RATS and 1837 VATS segmentectomies [29]; we found a non-significantly lower risk with VATS (adjusted HR = 0.9, 95% CI: 0.659–1.229; *P* = 0.51).

According to the AJCCv7 stage-specific survival rates published by Yun *et al.* [18], our expected 2-year survival rate was 94.0%, excluding stages 0 and IV. We observed a 2-year survival rate of 95.7% (95% CI: 88.4–98.8%). This rate is compatible with the expected rate (94.0%), but it is also compatible with much lower (88.4% at worst) or better (98.8% at best) rates. Therefore, we cannot precisely make conclusions about the equivalence of the midterm outcome.

### Limitations 

Our study has some limits: It is a case series, without a comparative group, with a retrospective analysis of prospectively collected data but with quality control by a data manager. This series is too small to provide stage-specific 2-year OS rates, and the follow-up period is too short to provide a 5-year OS rate, even when pooling all stages. RATS is still criticized because of its cost and oncological results compared with those from VATS. Le Gac *et al.* published in February 2020 a study carried out in our institution about the cost of the learning curve for RATS segmentectomies; they concluded that inexperienced surgeons may have higher procedural costs related to consumable medical materials and operating times. The learning curve for the lung segmentectomy was completed at more than 30 RATS procedures [30]. Currently, the same team is making a comparative study between VATS and RATS for mortality, morbidity, oncological results and operative costs.

## CONCLUSION

RATS segmentectomy is promising, with good short-term and midterm outcomes. Imaging-guided sublobar RATS resection seems to be useful for surgeons to perform oncological resections, especially for early-stage NSCLC. Although small, our series is one of the largest to date and may contribute to further meta-analyses.

**Conflict of interest:** Jean Marc Baste is a proctor for Intuitive Surgical, Johnson and Medtronic. All other authors who have contributed to this paper have no conflicts of interest to declare. 

### Author contributions

**Zied Chaari:** Data curation; Formal analysis; Writing—original draft. **François Montagne:** Data curation; Writing—review & editing. **Matthieu Sarsam:** Writing—review & editing. **Benjamin Bottet:** Writing—review & editing. **Philippe Rinieri:** Supervision. **Andre Gillibert:** Formal analysis; Methodology. **Jean Marc Baste:** Conceptualization; Supervision; Writing—review & editing.

### Reviewer information

Interactive CardioVascular and Thoracic Surgery thanks Clemens Aigner and the other, anonymous reviewer(s) for their contribution to the peer review process of this article.

## ABBREVIATIONS

3D: Three-dimensional
CIs: Confidence intervals
CT: Computed tomography
HR: Hazard ratio
ICG: Indocyanine green
IQR: Interquartile range
NSCLC: Non-small-cell lung cancer
OS: Overall survival
RATS: Robotic-assisted thoracic surgery
VATS: Video-assisted thoracoscopic surgery
